# Mothers' and fathers' views on the importance of play for their children's development: Gender differences, academic activities, and the parental role

**DOI:** 10.1111/bjep.12520

**Published:** 2022-05-25

**Authors:** Gillian M. Waters, Georgina R. Tidswell, Eleanor J. Bryant

**Affiliations:** ^1^ University of Bradford Bradford UK

**Keywords:** academic activities, gender, learning, parental role, parents, play

## Abstract

**Background:**

Play is a main driver of children's cognitive and social development and is crucial for educational success (*Paediatrics*, **119**, 2007 and 182). In recent years, however, parents and schools are under pressure to prioritize academic targets over play.

**Aims:**

The current research investigated parents' views about three aspects of their children's play and academic activities.

**Sample:**

Predominantly highly educated UK parents (109 mothers and 49 fathers) were recruited via social media.

**Method:**

Participants were asked to complete an amended online version of the Preschool Play and Learning Questionnaire (*International Journal of Behavioral Development*, **28**, 2004 and 97). The questionnaire consisted of 25 items covering three themes: the importance of play for children's development, the importance of academic activities, and the importance of parents' role in their children's development. The independent variables were the gender of the parent, the gender of their child, and the age group of their child (4–7 years, or 8–11 years).

**Results:**

Parents rated play higher than academic activities or their own roles, but the difference was not noteworthy. However, fathers rated academic activities and the parents' role significantly higher than mothers did. In addition, parents of girls rated academic activities and their own role significantly higher than parents of boys.

**Conclusions:**

The findings of the current research highlight gender divisions between parents and towards boys and girls regarding the importance of education. Gender roles appear to influence the way parents think about the academic activities their children partake in.

## BACKGROUND

Play is important for academic success: pre‐school‐aged children's interactions during playtime require the social, cognitive, and language skills, which aid learning (Dickinson & Tabors, 2001; LaForett & Mendez, 2016; Pellegrini & Galda, 1993) and improve readiness for school (Coolahan et al., 2000). Complex pretend play, in particular, helps pre‐school and young school‐aged children learn early academic skills (e.g. numbers, number order, letters, and vocabulary) (Karpov, 2005) and is linked to the development of narrative thinking, problem‐solving, and decision‐making in middle childhood (Singer & Singer, 2006). Even in adolescence and adulthood, play is linked to creativity and scientific thinking: we might view Isaac Newton and Alexander Fleming as great scientists, but they saw themselves as “playing” (Armstrong, 2006).

Play used to be considered a fundamental part of young children's education (Walsh et al., 2017) but play‐based practice is gradually being replaced with more structure, classroom instruction, and standardized assessments, in the United States (Bassok et al., 2016; Bowdon & Desimone, 2014; Markowitz & Ansari, 2020; Miller & Almon, 2009; Nicolopoulou, 2010), Australia (Harman & Harms, 2017), and the United Kingdom (Wood, 2015). Parents also often emphasize early educational training over play (Parmar et al., 2004; Parmar et al., 2008) and even children see play as less important than schoolwork (O'Gorman & Ailwood, 2012). Indeed, playtime is frequently used by parents and teachers as a reward for good behaviour or for completing the “work” of learning (Munn et al., 2009) rather than a vital part of children's development (Duncan & Lockwood, 2008).

Parental attitudes concerning the importance of play seem to fall on a spectrum. At one end, the focus is on academic achievement with little time put aside for play (Johnson et al., 2005) and at the other end are parents who prioritize child‐oriented play (Shiakou & Belsky, 2013) and believe that it is a valuable developmental activity (“Play Support”) (Fogle & Mendez, 2006). Parents who understand the significance of play tend to participate in play activities and encourage them (Farver & Howes, 1993; Haight et al., 1997). Crucially, the children of parents who treat play as valuable are more likely to have higher cognitive abilities, better social skills, and show greater independence (Lin & Yawkey, 2014; Parker et al., 1999). Unstructured free play (e.g. dressing up) is the most beneficial for children's emotional and cognitive development, as it minimizes stress, allows time for reflection, and builds resilience (Wiium & Safvenbom, 2019). However, for most children, their spare time is controlled rather than “free” (Fisher et al., 2008; Hofferth, 2009). Many parents view goal‐oriented pursuits as more important than unstructured play (Fisher et al., 2008), so children spend their spare time watching screens (Singer et al., 2009), “playing” with digital devices, or taking part in organized after‐school activities (Nordbakke, 2019).

Mothers' attitudes tend to be the focus of research in the area of play, probably because of the time they tend to devote to infants and young children (Morris, 2013). Yet, fathers often spend more time playing with their children than mothers (Brown et al., 2011; Craig, 2006). Father–child play can be an important part of family life (Yeung et al., 2001) and can contribute greatly to children's development (for a review see Amodia‐Bidakowska et al., 2020). Fathers are studied on average three times less than mothers (Cabrera & Roggman, 2017); yet, the proportion of men looking after their children full‐time is growing, with numbers in the United Kingdom increasing tenfold between 2000 and 2010, and one in seven fathers being the main childcare provider (Chalabi, 2013). Parents' own roles, as instigators and encouragers of play, may be evolving following these shifts in parenting roles and responsibilities and more research about fathers' views on play is clearly needed (Bornstein et al., 2010).

There are differences between parent's play, with fathers tending towards “rough‐and‐tumble” play (especially with their sons) (Lindsey & Mize, 2001; StGeorge & Freeman, 2017) and mothers more likely to engage in structured play (John et al., 2012). When mothers do engage in physical play, it is also more likely to be with their sons, or with older children (Schoppe‐Sullivan et al., 2013). Mothers tend to communicate more with their daughters than sons during play (Elkind, 2010), while fathers are more likely to sing to their daughters (Mascaro et al., 2017). Mothers and fathers also seem to vary in their views about the educational benefits of play (Gleason, 2005) and the amount of pressure they put on their children to achieve academically (Warash et al., 2000), although the literature is inconclusive (Warash et al., 2016).

Parents also treat their sons and daughters differently: they are more likely to engage in pretend play with girls than boys (John et al., 2012) and tend to discourage aggression and encourage prosocial behaviour in girls' play, but encourage more responsibility and turn‐taking games with boys (Power & Parke, 1986). Such gender‐stereotypical behaviour tends to support the socialization and conformity of children to what is “normal” in their society (Bornstein et al., 2016). For example, in the United States, educational activities are considered more important for young girls as they age, than for young boys (Warash et al., 2016). Across many cultures, the belief that free time should be filled with scheduled activities seems more important for parents of girls than for parents of boys (Knoop & Jensen, 2003). However, most extant research on gendered attitudes towards play and education focuses on pre‐school age or adolescence rather than those in young (4–7 years) or middle childhood (7–11 years).

In summary, play is an essential factor in children's development, and is crucial for academic success. It is important to study parents' beliefs about the purpose and value of play as they can influence: the type of play their children engage in; the amount of time children spend playing; whether they (the parents) get involved in playing with their children. Valuing play can have a direct and positive impact on the development of children's socio‐emotional competence and a variety of cognitive skills; however, parents are also under pressure to emphasize education. Opinions about the value of play often seem based on social expectations of intellect and academic attainment and can vary according to gender (as well as social and cultural expectations) (Parmar et al., 2004). Most research around play tends to focus on mothers' views and there is a need for more exploration of fathers' attitudes, especially in the United Kingdom where there has been a huge increase in the number of stay‐at‐home fathers. In addition, most research about play tends to focus on infants or pre‐schoolers and less is known about parents' opinions about older children. Consequently, the aim of the present research was to clarify UK mothers’ and fathers' views on the importance of play and learning for their 4‐ to 11‐year‐old sons and daughters.

## METHOD

### Participants

A power analysis using G*Power (Faul et al., 2007) with α = .05 and power = .80 suggested that a total sample size of at least 135 participants was required to detect a medium effect size (f = .25). An online survey advertised on social media recruited 157 participants from the United Kingdom (49 fathers and 108 mothers) who answered questions about one child in their family. There were 82 participants who responded about a child in the 4‐ to 7‐year age group (41 girls and 41 boys), and 75 participants who responded about a child in the 8‐ to 11‐year age group (35 girls and 40 boys). Mothers and fathers from the same family unit were not permitted to respond. Most participants were highly educated: 40.5% had a first degree; 17.1% had a master's degree; 6.3% had a PhD; and 10.1% had professional training.

### Design

The Pre‐school Play and Learning Questionnaire (PPLQ) (Parmar et al., 2004) was chosen, as it incorporated parental views on the importance of: education, play, and the parents' role in children's development. It was adapted for a UK population and an older age range of children. An independent design was used with three independent variables: gender of child (male; female), age group of children (4–7 years; 8–11 years), and gender of parent (male; female). The three dependent variables were the sub‐categories of the PPLQ: the importance of academic activities for development; the importance of play for development; and the parents' role in play and development (Parmar et al., 2004).

### Materials

The Preschool Play and Learning Questionnaire (PPLQ) (Parmar et al., 2004) contains 25 items on a seven‐point Likert scale (rated from “strongly disagree” to “strongly agree”) and includes 10 statements endorsing the importance of play for development (e.g. “play is essential for the development of children”); seven endorsing the importance of academic activities for development (e.g. “it is very important for children to work hard, mostly on skills such as literacy and numeracy”); and eight concerning parents' role in play and development (e.g. “parents should help children in developing creativity and imagination”). Questions were randomized and counterbalanced to prevent order effects.

The PPLQ was adapted so that it would be more appropriate for UK participants and an older age range of children: “Academics” was changed to “skills such as literacy and numeracy”; “math” was changed to “maths”; “pre‐schoolers” was changed to “children”; “for studies” was changed to “to study”; “watching TV and videos” was changed to “screen time” (including TV and mobile media devices such as iPads); “learn computers” was changed to “learn about technology”. Three questions were also added to the beginning of the questionnaire to collect the following information: participant role (mother or father), child gender, and child age group (4–7 years or 8–11 years).

### Procedure

An advert and link were posted on social media. Participants read a brief description of the study, and, if they chose to click on the link, they were taken to the online survey. No incentive was offered. Participants completed the required demographics and then each statement in turn (order counterbalanced) by choosing one option on a seven‐point Likert scale (from “strongly agree” to “strongly disagree”). Participants were asked to respond to the statements while considering just one of their children (if they had more than one) at their current age.

## RESULTS

Parents' ratings of 25 items were categorized according to their content (face validity) into three sub‐themes suggested in previous research (Parmar et al., 2004): the importance of play for development (10 items); the importance of academic activities for development (7 items) and parents' role in play and development (8 items) (see Table 1 for means and standard deviations).

**TABLE 1:** Mean scores (± SD) and percentages for the importance of play, academic activities, and parents' role in children's development

Importance of:	Total mean (SD)	Mothers (*n* = 108) Mean (SD)	Fathers (*n* = 49) Mean (SD)
Play (max possible score: 70)	56.41 (4.93)	56.45 (5.10)	56.31 (4.60)
Mean as a percentage of max possible score	81%	81%	80%
Academic activities (max possible score: 49)	29.53 (4.83)	29.09 (4.90)	30.67 (4.53)
Mean as a percentage of max possible score	60%	59%	63%
Parents' role (max possible score: 56)	40.18 (4.92)	39.62 (5.15)	41.41 (4.17)
Mean as a percentage of max possible score	72%	71%	74%

A multiple analysis of variance (MANOVA) was carried out using the three independent variables: gender of child (male; female), age group of child (4–7 years; 8–11 years), gender of parent (male; female) and the three dependent variables: the importance of academic activities for development; the importance of play for development; and parents' role in play and development (from now on to be referred to respectively as “academic activities”, “importance of play”, & “parents’ role”). Overall, there was a significant main effect for the gender of the parent *F*(3,142) = 3.04, *p* = .031, η_ρ_^2^ = .06, with fathers (*M* = 128.50; *SD* = 10.36) rating items as more important overall than mothers (*M* = 125.20; *SD* = 11.60). There was also a significant main effect for child gender (*F*(3,142) = 3.17, *p* = .026, η_ρ_^2^ = .063), with parents of girls (*M* = 128.68; *SD* = 11.26) rating items as more important overall than parents of boys (*M* = 123.87; *SD* = 10.88). There was no difference in the overall scores given for the two age groups of children.

The MANOVA analysis of the three sub‐themes revealed that academic activities were rated as more important by fathers (*M* = 30.67, *SD* = 4.53) than mothers (*M* = 29.09, *SD* = 4.90), *F*(1,144) = 5.59*, p* = .019, η_ρ_^2^ = .037. The parents' role was also rated as more important by fathers (*M* = 41.41, *SD* = 4.17) than mothers (*M* = 39.62, *SD* = 5.15) *F* (1,144) = 7.45, *p* = .007, η_ρ_^2^ = .049 (see Table 1 for Means and SDs). Parents of girls (*M* = 30.33, *SD* = 5.13) rated academic activities as more important than parents of boys (*M* = 28.78, *SD* = 4.44) (*F*(1,144) = 4.61, *p* = .033, η_ρ_^2^ = .031). Parents of girls (*M* = 41.20, *SD* = 4.92) also rated the parents' role as more important than parents of boys (*M* = 39.21, *SD* = 4.75) *F*(1,144) = 8.90, *p* = .003, η_ρ_^2^ = .058. There were no effects of age, and no other main effects or interactions.

## CONCLUSIONS

We sought to investigate UK mothers’ and fathers’ attitudes towards their children's play. The findings suggest that both parents consider play to be important for their children's development, but not significantly so. Parents who value play have been shown to engage in more play with their children (Haight et al., 1997) which is beneficial to the child's play level (Lang, 2009) and to the parent–child relationship (Ginsburg, 2007), so its continued importance to parents is encouraging. However, just because parents acknowledge the importance of play, it does not necessarily equate to them playing with their children or prioritizing play for them (Coo et al., 2020; Shiakou & Belsky, 2013).

We note that our sample was highly educated and consequently likely to be of a high socio‐economic status (SES). There may also have been low ethnic diversity (although exact demographics were not reported). Lower SES groups are associated with less time spent in parent–child educational activity (Aram et al., 2013; Kluczniok et al., 2013; Neumann, 2016), and less pretend play (Karnik & Tudge, 2010), while ethnic minority parents can hold different views about parental involvement in play (DiBianca Fasoli, 2014). However, while low SES families historically possessed fewer educational resources than higher SES groups (Aram & Levin, 2001), in the United Kingdom (at least), they are now more likely to have mobile technology (Ofcom, 2014), which can be a valuable platform for children's learning, especially when parents guide use (Neumann, 2018). Future research could investigate recent changes in access to technology across SES and ethnic minority groups in the United Kingdom, and the impact it has on children's play.

While Parmar et al. (2004)’s focus was on cultural differences in attitudes towards play between parents, our focus was on gender differences. We found that fathers and mothers' views varied significantly across most of our measures (although there were far fewer fathers who responded). First, fathers believed that academic activities were more important for their children's development than mothers did, which supports other research concerning fathers' emphasis on education (Warash et al., 2016). It is possible that fathers think about their child's future development more than they consider normal day‐to‐day childcare (Bartlett et al., 2018) and that our findings reflect the increase in stay‐at‐home fathers in the United Kingdom (and subsequent probable increase in father–child play). Family structure can also have an impact on the amount of play and extra‐curricular activities in the household. For example, children with many siblings spend more time playing, whereas children of single mothers spend more time in structured sport activities (Hofferth & Sandberg, 2001). It seems prudent to further investigate the changing structure of UK families and how this might impact children's play.

Fathers also rated the parents' role overall as more important than mothers did. There has been a gradual, almost global, increase in men's involvement in childcare over recent years (Hook & Wolfe, 2012); however, this does not mean that responsibilities are equally distributed. Parents may share the responsibility for “fun” play activities equally between them, but mothers tend to provide most educational activities (Keizer et al., 2020) and supervision of homework (Morgan et al., 2009; Morris, 2013). Fathers still tend to be seen as the “breadwinners” of the family, while mothers routinely take on most of the childcare (Chesley & Flood, 2017), even in higher earning households and more egalitarian countries (Craig & Mullan, 2011). Despite the changing gender distribution of stay‐at‐home parents, it may be that stereotypical roles continue to dominate.

Most interestingly, we found that parents of girls rated academic activities as more important for their children's development than parents of boys. They believed that their daughters should learn school‐related skills at home every day and have more books than toys, while these matters were not so important for their sons. Other research has found that parents encourage schoolwork in girls more than boys (Varner & Mandara, 2013); and mothers have higher educational expectations of girls than boys (Raley & Bianchi, 2006). Our findings may explain why in the United Kingdom, at least, girls currently outperform boys academically (Erin et al., 2020; GOV.UK, 2021).

Besides educational expectations, our findings show that parental involvement in their child's development was more important to parents of girls than parents of boys. Other research has found that parents give toddler girls more assistance in physical tasks than toddler boys, regardless of their abilities (Morrongiello & Dawber, 1999) and are more likely to initiate pretend play with pre‐school girls than boys (Lindsey et al., 1997). Fathers also tend to be more involved in their son's lives (Raley & Bianchi, 2006) and ignore their daughter's play initiations (Lindsey, Cremeens, & Caldera, 2010) while girls have more involvement from parents than boys (Carter & Wojtkiewicz, 2000) but are controlled by parents more (Smetana & Daddis, 2002; Van Lissa et al., 2019). The inequality in how we treat our sons and daughters' merits continued investigations. Specifically, it would be interesting to question non‐nuclear families (single, same sex, blended, etc.) and those who may not support traditional stereotypical gender roles about their views on their roles as parents.

Despite other research suggesting that parental support for play decreases (at a trend level) with child age (Warash et al., 2016) while encouragements in academic activities increases (Parmar et al., 2004), we found no difference between children's age groups. However, this could simply be due to the similarity between our age groups: in the United Kingdom, children between 4 years and 11 years usually attend the same schools. Intriguingly, our mothers' and fathers' views on the importance of play for development were almost identical. This is surprising as a recent US survey of parents of pre‐school children found that mothers placed a higher value on play than fathers (Warash et al., 2016). However, the children in our study were older and their play experiences would happen outside of school hours, in contrast to pre‐schoolers. As Western middle‐class fathers spend more time playing with their children than mothers (Craig, 2006), especially at weekends (Hook & Wolfe, 2012), this may explain the similarity between our parents' responses.

Our parents’ views on the order of importance (play, then parents’ role, then academic activities) place them in line with similar Euro‐American parents (Parmar et al., 2004). Nevertheless, parents' support of play does not always translate into a prioritizing of play in their children's lives (Shiakou & Belsky, 2013). For example, Greek parents rated play as more important than academic activities when given the PPLQ (Parmar et al., 2004), but their children spent more time on academic activities after school than they did for playing. We conclude that parents might value play and want their children to spend more time playing, but the pressure of academic achievement wins in the end (Sigel et al., 2014).

Our findings provide new information about UK parents’ opinions about play; however, there were some limitations and methodological issues that must be noted. The sample size was small, and there were fewer fathers than mothers, which suggests that our findings regarding parent gender differences should be interpreted with some caution. Our sample was also highly educated and likely the majority were middle‐class and Caucasian. The lack of ethnic diversity and limited range of SES mean that our results are not generalizable to the wider population of the United Kingdom. Although we acknowledge the usefulness of the PPLQ (Parmar et al., 2004), it is a self‐report survey, and answers could reflect social desirability. Additionally, some questions on the PPLQ concerning “the parents’ role” appear to be academically orientated (e.g. “parents should teach their children school‐related skills at home every day”), which blurs the boundary between the two sub‐themes. Other items on the PPLQ seem too broad (e.g. what type of development is being referred to in “children develop best through play”?) and may have led to participant confusion or confirmation bias. The adaptations we made to the questions (although minor) were not assessed for ecological validity, so it is possible that the adapted study was not as dependable as the original. The implications are that reworking and revalidating the PPLQ could create a much broader all‐encompassing survey to allow greater insights into parents' attitudes towards play.

In conclusion, the current study investigated parental views about their 4–11‐year‐old children's play and education. While playtime was considered important for development, it was not deemed to have significantly more value than educational pursuits. Parents rated academic activities as being more important for their daughters than sons, suggesting an inequality and higher expectations for girls to focus on education. Additionally, we found that fathers put greater emphasis on academic activities than mothers, confirming the differing opinions between parents and supporting existing research (Warash et al., 2016). Our findings should be interpreted with some caution; nevertheless, they offer a snapshot of middle‐class UK parents' views about the importance of play and open several avenues for future research.

## CONFLICT OF INTEREST

All authors declare no conflict of interest.

## AUTHOR CONTRIBUTIONS

**Gillian M. Waters:** Supervision; writing – review and editing. **Georgina R. Tidswell:** Conceptualization; data curation; formal analysis; investigation; project administration; writing – original draft. **Eleanor J. Bryant:** Validation; writing – review and editing.

## Data Availability

The data that support the findings of this study are available on request from the corresponding author. The data are not publicly available due to privacy or ethical restrictions.
