# Supplementary material for: Mothers' and fathers' views on the importance of play for their children's development: Gender differences, academic activities, and the parental role
Source: Br J Educ Psychol. 2022 May 25;92(4):1571–81. doi: 10.1111/bjep.12520 (PMC9790630; doi:10.1111/bjep.12520)
Supplement: Supplementary file 1 [file BJEP-92-1571-s001.docx]

## *Supporting Information.*

## *Play and Learning Questionnaire (adapted from PPLQ Parmar et al. 2004)*

Instructions: The following is a list of statements about children's play and learning. Please indicate how much you agree or disagree with each statement.

|  | **Strongly Agree** | | | | | | | | | |
| --- | --- | --- | --- | --- | --- | --- | --- | --- | --- | --- |
|  | **Agree** | | | | | | | |  | |
|  | **Somewhat Agree** | | | | | | |  | |  |
| **Please indicate your level of agreement** | **Neutral** | | | | | |  |  | |  |
| **with each statement.** | **Somewhat Disagree** | | | | |  |  |  | |  |
|  | **Disagree** | | | |  |  |  |  | |  |
|  | **Strongly Disagree** | | |  |  |  |  |  | |  |
|  | |  |  |  |  |  |  |  | |  |
| 1. Play is essential for the development of children. ……….. | |  | 1 | 2 | 3 | 4 | 5 | 6 | | 7 |
| 1. It is important for children to have toys such as memory games and puzzles that help with their cognitive development. | |  | 1 | 2 | 3 | 4 | 5 | 6 | | 7 |
| 1. Parents should teach their children school-related skills at home every day. ………………………………………… | |  | 1 | 2 | 3 | 4 | 5 | 6 | | 7 |
| 1. Children should learn social skills primarily through play.……………. | |  | 1 | 2 | 3 | 4 | 5 | 6 | | 7 |
| 1. Children should have more books than toys. …………………… | |  | 1 | 2 | 3 | 4 | 5 | 6 | | 7 |
| 1. Parents should be playmates to their children. …………………. | |  | 1 | 2 | 3 | 4 | 5 | 6 | | 7 |
| 1. Play is an important activity for children to learn to be independent. ………………………………………………………… | |  | 1 | 2 | 3 | 4 | 5 | 6 | | 7 |
| 1. It is important for children to have a "play room" or "play space" at home. ……………………………………………………... | |  | 1 | 2 | 3 | 4 | 5 | 6 | | 7 |
| 1. Play should always have some educational value for children ... | |  | 1 | 2 | 3 | 4 | 5 | 6 | | 7 |
| 1. Parents should initiate play with their children………….……. | |  | 1 | 2 | 3 | 4 | 5 | 6 | | 7 |
| 1. TV and mobile media devices (e.g. iPad) can be important resources for children's learning. | |  | 1 | 2 | 3 | 4 | 5 | 6 | | 7 |

|  | **Strongly Agree** | | | | | | | | | |  |
| --- | --- | --- | --- | --- | --- | --- | --- | --- | --- | --- | --- |
|  | **Agree** | | | | | | | | |  | |
|  | **Somewhat Agree** | | | | | | | |  |  | |
| **Please indicate your level of agreement** | **Neutral** | | | | | | |  |  |  | |
| **with each statement.** | **Somewhat Disagree** | | | | | |  |  |  |  | |
|  | **Disagree** | | | |  | |  |  |  |  | |
|  | **Strongly Disagree** | | |  | |  |  |  |  |  | |
|  | |  |  |  | |  |  |  |  |  | |
| 1. Children develop best through play. ……………………………... | |  | 1 | 2 | | 3 | 4 | 5 | 6 | 7 | |
| 1. Children should have toys that are just for fun. ……………. | |  | 1 | 2 | | 3 | 4 | 5 | 6 | 7 | |
| 1. It is very important for children to work hard, mostly on skills such as literacy and numeracy. …………………………………………. | |  | 1 | 2 | | 3 | 4 | 5 | 6 | 7 | |
| 1. Parents should let their children learn about technology as early as possible. …………………………………………………... | |  | 1 | 2 | | 3 | 4 | 5 | 6 | 7 | |
| 1. Parents should only play with their children when children ask them to do so. ……………………………………………………… | |  | 1 | 2 | | 3 | 4 | 5 | 6 | 7 | |
| 1. It is important for children to have a stimulating environment with lots of toys. …………………………………………………… | |  | 1 | 2 | | 3 | 4 | 5 | 6 |  | |
| 1. Children should have some structured time at home each day for   school work even if there is no homework ………………………………………………... | |  | 1 | 2 | | 3 | 4 | 5 | 6 | 7 | |
| 1. Children should not be pushed to study if they are not interested. …………………….………………….. | |  | 1 | 2 | | 3 | 4 | 5 | 6 | 7 | |
| 1. Parents should supervise their children’s homework every night. ………………. | |  | 1 | 2 | | 3 | 4 | 5 | 6 | 7 | |
| 1. Screen time (e.g. watching TV and media devices) should be kept to a minimum. ………….. | |  | 1 | 2 | | 3 | 4 | 5 | 6 | 7 | |
| 1. Parents should help children in developing creativity and imagination. ………. | |  | 1 | 2 | | 3 | 4 | 5 | 6 | 7 | |
| 1. Children should have lots of time to play and have fun every day. …………. | |  | 1 | 2 | | 3 | 4 | 5 | 6 | 7 | |
| 1. Children should have toys that help them learn school-related skills such as maths and science. ………………………………… | |  | 1 | 2 | | 3 | 4 | 5 | 6 | 7 | |
